# Intraarticular hemorrhage due to bevacizumab in a patient with metastatic colorectal cancer: a case report

**DOI:** 10.1186/1752-1947-6-188

**Published:** 2012-07-09

**Authors:** Mukremin Uysal, Sema Sezgin Goksu, Hasan Senol Coskun, Burhan Savas, Mustafa Ozdogan, Hakan Bozcuk

**Affiliations:** 1 Department of Medical Oncology, Akdeniz University Faculty of Medicine, Antalya, Turkey

**Keywords:** Bevacizumab, Colorectal cancer, Synovial metastasis, Intaarticular hemorrhage

## Abstract

**Introduction:**

Bevacizumab is a monoclonal antibody against vascular endothelial growth factor. It is widely used in the treatment of metastatic colorectal cancer. It has some specific side effects including severe bleeding, wound healing problems, gastrointestinal perforation, proteinuria and hypertension.

**Case presentation:**

We present the case of a 65-year old Asian man with synovial metastasis of the knee who experienced intraarticular hemorrhage after bevacizumab treatment. He presented with monoarthritis of the left knee.

**Conclusion:**

Bevacizumab-related hemorrhage can cause serious morbidity and unusual sites of hemorrhage may be seen.

## Introduction

Colorectal cancer is the third most common cancer in both men and women. should be: "It is also the third most common cause of cancer-related deaths [1]. Survival rates have increased from 41% to 66% between 1950 and 2000. The main reason for this improvement is the use of effective cytotoxic therapies such as oxaliplatin and irinotecan. Targeted therapies beginning with bevacizumab in 2004 have improved the overall survival of patients with metastatic colorectal cancer up to two years [2].

Bevacizumab is the first angiogenesis inhibitor that binds and inactivates all isoforms of vascular endothelial growth factor (VEGF). It has some specific side effects including severe bleeding, wound healing problems, gastrointestinal perforation, proteinuria and hypertension. Hemorrhages are usually related to metastasis or primary tumor itself. No case of intraarticular bleeding due to bevacizumab has been reported previously in the literature. We report the case of a man with knee joint metastasis from colon adenocarcinoma who experienced intraarticular hemorrhage subsequent to bevacizumab treatment.

## Case presentation

**Figure 1:**
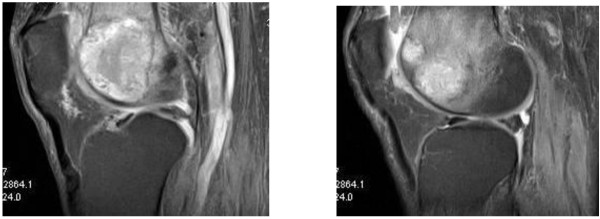
**Magnetic resonance image showing knee joint and synovial metastasis.** In short TI reversion proton density (STIR PD) sequences; sagittal plane images, there is a lesion in the femur medial condyle epiphyseal-metaphyseal line which has a peripheral hyperintense signal and shows edematous changes in the near bone. In the same sequences, there is an increase of synovial fluid in the suprapatellar pouch and at this location there is another poor hyperintense lesion (bone and synovial metastasis).

A 65-year-old Asian man was diagnosed with stage 3 colon cancer and treated with adjuvant fluorouracil and leucoverin. After six years he had a relapse with brain and lung metastates. After cranial metastasectomy, he was treated with full brain radiotherapy. Chemotherapy with capecitabine and bevacizumab was started (capecitabine 1000mg/m² twice a day one to 14 days, bevacizumab 7.5mg/kg on the first day, every 21 days). Oxaliplatin- or irinotecan-based chemotherapy could not be given due to his poor Eastern Cooperative Oncology Group (ECOG) performance score. The lung lesion was resected after partial response to six cycles of chemotherapy. He discontinued therapy and did not come to control visits. After three months he presented to our clinic with weight loss, weakness and pain in his left knee. His carcinoembryonic antigen was increased and positron emission tomography-computed tomography (PET- CT) revealed multiple liver, lung and bone metastasis. Bevacizumab and capecitabine was restarted at the same dose and frequency as he had received previously. After the first course of this regimen, he presented with monoarthritis in the left knee. Physical examination revealed tenderness, swelling and reduced mobility. Laboratory studies revealed a hemoglobin value of 10.2g/dL; white blood count of 7800/mm^3^ (Normal ratio 10,800) and C-reactive protein of 0.45mg/dl (Normal ratio <0.4mg/dL). The left knee was aspirated and yielded hemorrhagic fluid. He had no history of a traumatic event or use of warfarin, heparin or acetylsalicylic acid. He had no history of hemorrhagic diathesis. Coagulation parameters were normal. A magnetic resonance image (MRI) revealed synovial metastasis of the knee (Figure 1). Cytologic examination was not done and he refused to have an arthroscopic biopsy. Bevacizumab was stopped and palliative radiotherapy (3000cGy in 10 fractions) to the left knee region was administered to relieve the symptoms.

## Discussion

This is the first case of bevacizumab-related intraarticular bleeding reported in the literature. Intraarticular hemorrhage was associated with the synovial metastasis of the left knee. Synovial metastasis is a rare entity; it is usually seen in solid tumors. Only a few reported cases of metastatic adenocarcinoma to the synovium can be found in the literature [3-6]. The differential diagnoses of monoarthritis in a patient with cancer include inflammatory diseases such as gout, avascular necrosis and septic arthritis which could be complications of chemotherapy. Various rheumatic disorders may also be seen [7]. In this patient bleeding stopped after discontinuation of bevacizumab and he had palliative radiation therapy to the left knee. Surgical procedures such as palliative synovectomy are also possible. If these measures fail and joint instability causes significant pain external or internal fixation may be considered.

Bevacizumab is the first angiogenesis inhibitor that binds and inactivates all isoforms of VEGF. The most common side effects are hypertension, proteinuria, epistaxis, gastrointestinal symptoms and poor wound healing. Infrequent serious adverse events include gastrointestinal perforation, arterial thrombotic events and hemorrhage [8]. Severe and life-threatening (Grade 3 or 4) bleeding has occurred among three to four percent of the patients who received bevacizumab. Hemorrhages are usually related to metastasis or the primary tumor itself. Bleeding events usually appear as epistaxis, gastrointestinal bleeding, hemoptysis, vaginal bleeding and intracranial hemorrhage and occur five times more often in patients treated with bevacizumab [8]. The most common form of hemorrhage is mild epistaxis that lasts less than five minutes and usually resolves without medication. These bleedings do not require discontinuation of bevacizumab therapy [9].

The mechanism underlying bevacizumab-related bleeding is not fully understood. It is probably associated with the inhibition of VEGF signaling. VEGF stimulates endothelial cell proliferation, promotes endothelial cell survival and helps to maintain the vascular integrity. Inhibition of VEGF can reduce the regenerative capacity of endothelial cells [10]. However, endothelial cell defects alone cannot explain the life-threatening hemorrhages in patients receiving anti-VEGF therapy; damage of the major vessel wall by tumor erosion, necrosis, cavitation and other pathologic conditions may have a role [10].

## Conclusions

Bevacizumab-related hemorrhage can cause serious morbidity even mortality. Unusual sites of hemorrhage may be seen. Clinicians should examine the symptoms of monoarthritis carefully in patients treated with bevacizumab and should not continue the therapy until they rule out intraarticular hemorrhage.

## Consent

Written informed consent was obtained from the patient for publication of this case report and any accompanying images. A copy of the written consent is available for review by the Editor-in-Chief of this journal.

## Abbreviations

VEGF, Vascular endothelial growth factor; PET-CT, Positron emission tomography-computed tomography.

## Competing interests

The authors declare that they have no competing interests.

## Authors’ contributions

MU and HSC designed the work; MU wrote the paper. MU, HSC, SSG, BS, MO and HB followed up with the patient. MU and HSC reviewed the article. All authors read and approved the final manuscript.
